# Control of Estrus in Sheep: Protocol Architectures, Seasonal Effects, and Breed-Specific Reproductive Performance

**DOI:** 10.3390/ani16152383

**Published:** 2026-08-03

**Authors:** Amar Akermi, Atika Ouled Ali, Mounir Adnane, Aspinas Chapwanya

**Affiliations:** 1 Department of Biomedicine, Institute of Veterinary Sciences, University of Tiaret, Tiaret 14000, Algeria; amar.akermi@univ-tiaret.dz (A.A.); atika.ouldali@univ-tiaret.dz (A.O.A.); 2 Department of Clinical Sciences, Ross University School of Veterinary Medicine, Basseterre 00265, Saint Kitts and Nevis

**Keywords:** estrus synchronization, seasonal anestrus, photoperiodism, hormonal protocols, artificial insemination, sheep reproduction

## Abstract

Sheep play a crucial role in strengthening food security in different regions, where climates are often variable and resources can be limited. The importance of these animals includes nutrition and income. For sustainable production, manipulating estrous cyclicity is a pivotal, particularly in tropical and dry climate regions. Given the variation in reproductive performance in various breeds with seasons, and management practices, it is difficult to select effective protocols at all times. There is a need for in-depth knowledge on efficiency of available protocols. This review critically synthesizes current estrus synchronization strategies in sheep, integrating physiological principles with comparative evaluation of hormonal molecules, protocol architectures, and reproductive performance data. Progesterone-based systems remain the cornerstone of synchronization, particularly when combined with equine chorionic gonadotropin (eCG), because they simulate luteal activity and promote follicular recruitment during seasonal anestrus. However, prolonged progestogen exposure may induce persistent follicles and compromise oocyte quality, whereas short-term protocols (5–7 days) frequently achieve comparable or improved fertility while reducing hormonal exposure. Gonadotropin-releasing hormone (GnRH)-based approaches improve control of ovulation timing and are particularly useful for fixed-time artificial insemination (FTAI), although their effectiveness declines during deep anestrus without exogenous gonadotropic support. Prostaglandin-only systems provide cost-effective synchronization in cyclic ewes, but their reliability is limited outside the breeding season. Emerging innovations, including recombinant gonadotropins and nano-formulated hormone delivery systems, aim to enhance endocrine precision while addressing welfare, immunogenicity, and regulatory concerns associated with conventional eCG use. Across studies, variation in outcomes reflects the complex interaction among endocrine regulation, follicular dynamics, luteal competence, breed-specific ovarian responsiveness, and seasonal neuroendocrine suppression. Therefore, current evidence suggests that synchronization success depends less on protocol complexity than on physiological compatibility with breed characteristics, seasonal reproductive status, and production objectives. Future research on precision endocrine modulation, reduced hormonal exposure, ethical standardized outcome reporting is warranted.

## Simple Summary

Controlling the timing of estrus (heat) in sheep is important for farmers to manage breeding, improve productivity, and use artificial insemination. This review compares different hormonal methods used to synchronize estrus. Progesterone-based treatments work well, especially when combined with another hormone called eCG, because they mimic the natural cycle and help follicles grow. However, using progesterone for too long (12–14 days) can lead to older, lower-quality eggs. Shorter treatments (5–7 days) often give similar or better results while using less hormone. Hormones like GnRH help control the exact time of ovulation and are useful for fixed-time artificial insemination, but they work best during the normal breeding season. Prostaglandin treatments are cheap and effective only in sheep that are already cycling. Emerging technologies, including recombinant hormones and experimental nano-formulated products, show promise in preliminary studies, although further validation across diverse production systems is required. Overall, the best method depends on the sheep breed, the season, and the farm’s goals. Future work should focus on fine-tuning hormone doses, reducing drug use, and standardizing how results are reported.

## 1. Introduction

Efficient reproductive management is central to sustainable sheep production, directly influencing flock productivity, genetic progress, and economic return [1]. Because profitable sheep production relies on the number of lambs weaned per 100 ewes bred per year, estrus synchronization has become a powerful tool in sheep husbandry [2,3]. In seasonal breeding species such as sheep, photoperiod-driven cyclicity limits lambing to the spring season. Ability to control the timing of ovulation and mating is therefore fundamental to modern flock management.

Estrus manipulation enables concentrated lambing, improved labor organization, better alignment of feed resources with nutritional demand, and the efficient implementation of assisted reproductive technologies such as artificial insemination (AI) and fixed-time artificial insemination (FTAI) [4]. In addition, this allows accelerated genetic improvement through the dissemination of superior sires and facilitates embryo transfer programs. The adoption of these hormonal strategies has been particularly widespread in South American sheep production systems, where highly seasonal breeds predominate and synchronization protocols represent an essential tool for maintaining reproductive activity during the non-breeding season. In countries such as Brazil, Uruguay, Argentina, and Chile, estrus synchronization combined with fixed-time artificial insemination has enabled producers to concentrate lambing, improve genetic progress, and enhance the profitability of extensive and intensive production systems alike [1,4,5]. This regional context underscores the practical importance of optimizing synchronization protocols for diverse breed types and seasonal conditions. Recently, numerous hormonal strategies have been developed to manipulate the hypothalamic-pituitary-gonadal axis, primarily through progestogens, prostaglandin F_2_α (PGF_2_α) and its analogs, equine chorionic gonadotropin (eCG), and Gonadotropin-Releasing Hormone (GnRH) analogues administered alone or in combination [4,6]. Among these, progestogen-based protocols supplemented with eCG and/or PGF_2_α remain the most widely used across breeding seasons [7]. Despite these being widely adopted, fertility still varies considerably depending on season, breed, and management conditions. A meta-analysis of 24 studies comprising 78 treatment groups and 1934 sheep showed pregnancy rates ranging from 90.37% for progesterone (P4)+eCG during the breeding season to 59.36% for the same protocol during the non-breeding season, while P4+PGF_2_α achieved 69.77% outside the breeding period [4]. Furthermore, comparisons between long-term (12–14 day) and short-term (5–7 day) progestogen protocols revealed no significant difference in pregnancy rate (effect size 0.07; I^2^ = 47.85%), suggesting that protocol duration alone does not determine fertility outcomes [3]. It seems that breed, nutritional status, environmental conditions, and protocol play a key role and therefore limit universal recommendations for all production systems [3,4].

The practical application of synchronization protocols is further complicated by the diversity of sheep production systems. Intensive dairy breeds (e.g., East Friesian, Lacaune) are typically managed for year-round milk production and have been selected for reduced seasonality, whereas extensive meat breeds (e.g., Charollais, Suffolk, and many indigenous breeds) often retain strong seasonal reproductive patterns and are managed under lower-input systems. These divergent selection pressures and management contexts influence ovarian responsiveness, depth of seasonal anestrus, and the economic feasibility of different hormonal protocols. Therefore, a production-type perspective is essential when evaluating protocol efficacy and developing practical recommendations.

Increased awareness has now influenced reproductive management decisions. European organic farming regulation (EU 2018/848) prohibits hormonal treatments for reproductive control, encouraging reliance on non-hormonal approaches such as the male effect and precision estrus detection technologies [8]. However, system-dependent outcomes have been observed: integration of these approaches increased AI-sired progeny by 39–43% in organic systems but reduced it by 54% in conventional farms [9]. These findings highlight the complexity of replacing endocrine-based protocols without compromising productivity.

Given the diversity of available hormones, protocol architectures, and reported outcomes, a critical synthesis integrating endocrine mechanisms with field-level reproductive performance is required. This review therefore evaluates current estrus synchronization techniques in sheep, focusing on comparative mechanisms of action, protocol design, reproductive outcomes, and practical constraints. By aligning physiological principles with quantitative evidence, the objective is to provide a coherent framework for protocol optimization and to identify directions for future research in sheep reproduction.

## 2. Physiological Basis of Estrus and Seasonality in Sheep

Understanding synchronization protocols first requires examining the physiological mechanisms governing seasonal reproductive activity in ewes. Photoperiod-dependent regulation of the hypothalamic-pituitary-gonadal axis defines the endocrine constraints that synchronization strategies attempt to manipulate. Sheep are seasonally polyestrous animals in which reproductive activity is governed primarily by photoperiodic control of the hypothalamic-pituitary-gonadal (HPG) axis [10]. Decreasing day length increases melatonin secretion from the pineal gland, which modulates GnRH neuronal activity and consequently luteinizing hormone (LH) and follicle-stimulating hormone (FSH) secretion [11]. During the breeding season, ovulation requires sustained high-frequency GnRH pulse, reaching approximately 7.8 pulses per 6 h in the follicular phase [12]. In contrast, seasonal anestrus is characterized by a marked reduction in GnRH pulse frequency (<1 pulse per 6 h), despite maintenance of pulse amplitude, preventing initiation of the preovulatory GnRH surge [12]. Photoperiod-driven alterations in estradiol negative feedback sensitivity, mediated in part by dopaminergic pathways, further modulate LH pulse frequency under long-day conditions [11].

The expression of reproductive seasonality is modulated by latitude, breed, and nutritional status, factors that interact with photoperiodic signals to determine the depth and duration of seasonal anestrus. Latitude determines the amplitude of annual photoperiodic changes: sheep at higher latitudes experience greater daylength variation and consequently exhibit more pronounced seasonal reproductive cycles compared to those at lower latitudes [13]. For example, at 19° N latitude, Pelibuey ewes are capable of ovulating continuously throughout the year, whereas Suffolk ewes maintained under the same conditions display seasonal anestrous periods similar to those observed at latitudes greater than 35° [14].

The depth and duration of seasonal anestrus vary considerably among sheep breeds, reflecting genetic differences in the neuroendocrine response to photoperiod [15]. Breeds of British origin, such as the Suffolk, Hampshire, and Border Leicester, typically exhibit a pronounced and relatively short breeding season (autumn to early winter) followed by a deep and prolonged anestrus during spring and summer [16,17]. In contrast, breeds of Mediterranean origin, including the Chios, Merino, and Sarda, generally have longer breeding seasons and shorter anestrous periods, enabling reproductive activity to extend into spring [15,18]. For example, Chios ewes in Greece were found to have significantly shorter periods of anoestrus (109 ± 8 days) and anovulation (63 ± 8 days) compared to Serres ewes (178 ± 5 and 149 ± 6 days, respectively), with some Chios individuals exhibiting continuous ovulation throughout the year [15]. This reduced seasonality is thought to reflect differences in the photoperiodic synchronization of an endogenous reproductive rhythm rather than differences in the photoperiodic drive of reproductive transitions per se [19]. Under identical photoperiodic conditions, Galway ewes begin and end their breeding season earlier than Finnish Landrace ewes, suggesting breed-specific differences in how photoperiod entrains the endogenous circannual rhythm [19]. Similarly, Merino rams subjected to simulated Mediterranean photoperiods start and finish their reproductive seasons approximately two months earlier than Suffolk rams [19]. Tropical and subtropical breeds, such as the St. Croix, Pelibuey, and Blackbelly, display even further reduced seasonality, with some populations exhibiting continuous or near-continuous reproductive activity [14]. Notably, the OOS line, a composite of Dorset, Rambouillet, and Finnish Landrace breeding selected over ten years for spring fertility, has been shown to have one of the shortest seasonal anestrous periods reported for temperate breeds [17]. Such selection has significantly reduced seasonal anestrus in several breeds, demonstrating that the neuroendocrine mechanisms regulating seasonality are amenable to genetic modification [17]. At the molecular level, the melatonin receptor subtype 1A (*MTNR1A*) gene is the primary genetic determinant of reproductive seasonality in sheep [17,18]. Polymorphisms in *MTNR1A* have been associated with reduced seasonality and extended breeding seasons in multiple breeds [20,21]. Additionally, genome-wide association studies have identified other candidate genes, including *PAK1*, *CYP19A1*, and *PER1*, as likely contributors to seasonal reproductive variation [22]. These genetic differences in seasonality have direct implications for estrus synchronization: breeds with deeper anestrus may require more intensive hormonal support (e.g., higher eCG doses or GnRH-based protocols) to achieve acceptable fertility outside the breeding season, whereas less seasonal breeds may respond adequately to simpler or lower-dose protocols [15].

Nutrition also plays a significant modulatory role [23]. While only severe malnutrition can significantly extend the length of seasonal anestrus, the level of body fat reserves can delay the onset of seasonal anestrus, particularly in Mediterranean environments [23]. Nutritional supplementation can enhance gonadotropin secretion, but the response is breed-dependent: Merino rams exhibit reproductive responses to improved nutrition irrespective of time of year, whereas Suffolk rams respond to nutrition only when the hypothalamic reproductive centers are not inhibited by photoperiod [24]. Thus, latitude, breed, and nutrition collectively shape the expression of seasonal reproductive activity and must be considered when designing synchronization protocols.

The estrous cycle of the ewe averages 16–17 days and comprises a luteal phase dominated by progesterone secretion from the corpus luteum and a follicular phase characterized by increasing estradiol concentrations culminating in the preovulatory LH surge [10]. Ovarian folliculogenesis proceeds in successive waves, typically three to four per cycle, with each wave involving recruitment, selection, and dominance of antral follicles [10,25]. Following prostaglandin-induced luteolysis, the preovulatory LH surge occurs approximately 59 ± 4.7 h after functional regression of the corpus luteum, and ovulatory follicles generally arise from large antral follicles (approximately 5.1 ± 0.4 mm) present at luteolysis [26]. Progesterone plays a central regulatory role by suppressing GnRH/LH pulsatility during the luteal phase, preventing premature LH surges, and modulating uterine prostaglandin F_2_α secretion to coordinate luteal regression [10].

Importantly, seasonal anestrus does not represent ovarian quiescence. Follicular waves continue to emerge, and at least one large antral follicle (>5 mm) is typically present, capable of steroid secretion when stimulated [27]. However, a dissociation between morphological and functional dominance is evident in sheep: declines in estradiol production precede measurable reductions in follicular diameter, indicating that follicle size alone is not a reliable indicator of ovulatory competence [27]. Thus, seasonal infertility primarily reflects neuroendocrine suppression of GnRH pulsatility rather than an absence of follicular growth. A better understanding of the endocrine mechanisms governing seasonal reproductive activity provides the physiological framework upon which estrus synchronization strategies can be tailored. Because reproductive activity in ewes is regulated by photoperiod-driven modulation of HPG axis, synchronization protocols must aim to artificially reproduce or override key hormonal events controlling follicular dynamics, luteal function, and ovulation. By manipulating progesterone exposure, inducing luteolysis through prostaglandin F_2_α, or stimulating gonadotropin release with GnRH or eCG, these protocols attempt to temporarily bypass seasonal endocrine constraints and align follicular maturation and ovulation across groups of animals. Consequently, the practical architecture of synchronization programs directly reflects the physiological control points within the reproductive endocrine system.

## 3. Endocrine Events and Protocol Architectures for Estrus Synchronization in Sheep

Estrus synchronization in sheep relies on the strategic administration of exogenous hormones to manipulate luteal regression, follicular wave dynamics, ovulation timing, and behavioral estrus expression. The principal hormonal classes include progesterone and synthetic progestogens, eCG, GnRH and its analogs, PGF_2_α and its derivatives, and emerging recombinant or metabolic agents. These molecules are rarely applied individually but are integrated into structured synchronization protocols whose efficacy depends on season, ovarian status, and production objectives. The endocrine mechanisms and operational differences between the major hormonal systems are summarized in the following figures, while their comparative biological consequences and clinical performance are presented in the following tables.

### 3.1. Progesterone and Synthetic Progestogens

Progestogens exert negative feedback on the hypothalamic-pituitary axis, suppressing LH surge and preventing premature ovulation. Pharmacologically, progesterone and its synthetic analogs (fluorogestone acetate, FGA; medroxyprogesterone acetate, MAP) act as agonists at the progesterone receptor in the hypothalamus and pituitary, reducing GnRH pulse frequency and thereby suppressing LH secretion. This negative feedback maintains a luteal-phase endocrine environment and prevents the preovulatory LH surge. Several progesterone-releasing devices are commercially available for estrus synchronization in sheep, differing in hormone content, delivery system, and duration of action. The EAZI-BREED™ CIDR^®^ Sheep Insert consists of 0.3 g of natural progesterone molded within a silicone elastomer over a flexible nylon spine [28]. FGA sponges are impregnated with a synthetic progestogen at doses typically ranging from 20 to 45 mg, with some studies evaluating doses as low as 15–20 mg. Reducing the FGA dose from 40 to 20 mg does not significantly affect ovarian follicular dynamics, suggesting that lower doses may be equally effective [29]. MAP sponges contain 60 mg of synthetic progestogen and are typically inserted for 12–14 days [30]. While Controlled Internal Drug Release (CIDR) devices release natural progesterone and are associated with less vaginal irritation compared to sponges, FGA and MAP sponges remain widely used due to their lower cost and established efficacy [10,31].

**Figure 1:**
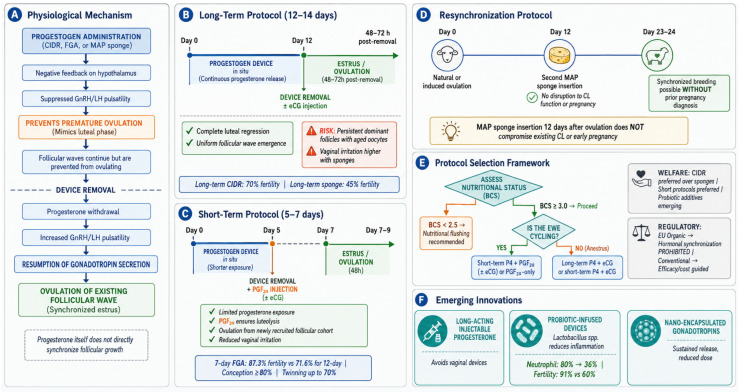
Progestogen-based estrus synchronization strategies in sheep. (**A**) illustrates the physiological mechanism common to all progestogen protocols: progestogen administration suppresses GnRH/LH pulsatility via negative feedback, preventing ovulation while follicular waves continue to emerge. Upon device removal, progesterone withdrawal restores gonadotropin secretion, allowing ovulation of the existing follicular wave in a synchronized manner. (**B**) depicts the long-term (12–14 days), which ensures complete luteal regression but carries risks of persistent dominant follicles and vaginal irritation. (**C**) shows the short-term protocol (5–7 days), which requires concomitant PGF_2_α administration for luteolysis and promotes ovulation from a newly recruited follicular cohort, yielding higher fertility. (**D**) illustrates resynchronization using MAP insertion 12 days after ovulation without compromising corpus luteum function or early pregnancy. (**E**) summarizes protocol selection framework incorporating nutritional status (BCS), cycling status, welfare considerations, and regulatory context. (**F**) highlights emerging innovations including injectable progesterone, probiotic-modified devices, and nano-encapsulated gonadotropins. BCS: body condition score, CIDR: Controlled Internal Drug Release (intravaginal progesterone device), CL: Corpus luteum, eCG: equine Chorionic Gonadotropin, EU: European Union, FGA: Fluorogestone Acetate, GnRH: Gonadotropin-Releasing Hormone, LH: Luteinizing Hormone, MAP: Medroxyprogesterone Acetate, P4: Progesterone, PGF_2_α: Prostaglandin F_2_ alpha.

The duration of progestogen exposure critically influences ovarian dynamics: prolonged treatment (12–14 days) permits emergence and persistence of dominant follicles, which may undergo luteinization or become atretic, potentially compromising oocyte quality [5,32]. Short-term protocols (5–7 days) allow synchronization of a newly recruited follicular cohort, provided that luteolysis is induced by concomitant PGF_2_α administration in cyclic ewes [33,34]. Withdrawal induces synchronized follicular development and estrus [31]. This mechanism underpins both long-term (12–14 day) and short-term (5–7 day) synchronization protocols. The endocrine mechanisms and operational differences between long-term and short-term progestogen-based synchronization strategies are summarized in Figure 1.

Historically, long-term treatments using CIDR devices or FGA or MAP sponges were considered standard, particularly for anestrous ewes [35]. Their extended duration ensures complete luteal regression and reliable synchronization across animals. However, prolonged progesterone exposure is mechanistically associated with persistent dominant follicles and potential oocyte aging [5,32]. In Hu sheep, no significant differences were observed between 11-day and 13-day FGA treatments in estrus response or lambing rate [36], suggesting limited benefit from extended duration.

Short-term protocols (5–7 days) represent a physiologically refined alternative (Figure 1B,C). A comparison of 7-day versus 12-day FGA treatments demonstrated similar estrus response but significantly higher fertility with the shorter protocol (87.3% vs. 71.6%) [33]. The improved outcome is attributed to ovulation of a newly recruited follicular cohort rather than aged persistent follicles. Effective short-term systems require concomitant PGF_2_α administration at or before device removal to ensure luteolysis in cyclic ewes [33,34].

Animal welfare considerations are increasingly influential in protocol selection. Intravaginal sponges have been associated with greater vaginal irritation and microbiota alterations compared to CIDR devices [31]. Long-acting injectable progesterone formulations are being explored as alternatives [37]. Additionally, early resynchronization strategies using a second MAP insertion 12 days after ovulation have been shown not to compromise corpus luteum function or early pregnancy, enabling consecutive breeding without prior pregnancy diagnosis [38,39,40] (Figure 1D). Although some studies were conducted in goats, the protocol architectures and endocrine principles, specifically the use of progestogens for resynchronization without compromising luteal function, are directly translatable to sheep and have informed similar approaches in ovine reproduction [39,40].

### 3.2. Equine or Human Chorionic Gonadotropins (eCG/hCG)

eCG possesses dual FSH/LH activity and a prolonged half-life (40–125 h), supporting follicular growth and ovulation, particularly during seasonal anestrus [41]. Biologically, eCG and hCG differ fundamentally in their source and production. eCG is a heavily glycosylated heterodimeric glycoprotein hormone produced by the endometrial cups, specialized fetal trophoblast cells that invade the maternal endometrium, in pregnant mares [42]. Secretion begins around days 36–40 of gestation, peaks between days 60 and 80, and declines gradually until approximately day 130 [41,42]. The hormone is traditionally extracted from the serum of pregnant mares (hence the former name PMSG: Pregnant Mare Serum Gonadotropin) [43]. In contrast, hCG is a polypeptide hormone produced by trophoblast cells of the human placenta, primarily by the syncytiotrophoblast [44]. Secretion begins soon after implantation, with levels doubling every 2–3 days and peaking at approximately 6–8 weeks of gestation [45]. Commercially, hCG is obtained from the urine of pregnant women [46]. Both hormones are heterodimeric glycoproteins composed of a common alpha subunit non-covalently linked to a hormone-specific beta subunit [42]. Structurally, eCG is unique among gonadotropins in that its beta subunit is identical to that of equine LH, which accounts for its potent LH-like activity [42]. These differences in biological source, glycosylation, and pharmacokinetics underpin their distinct applications in reproductive management.

**Figure 2:**
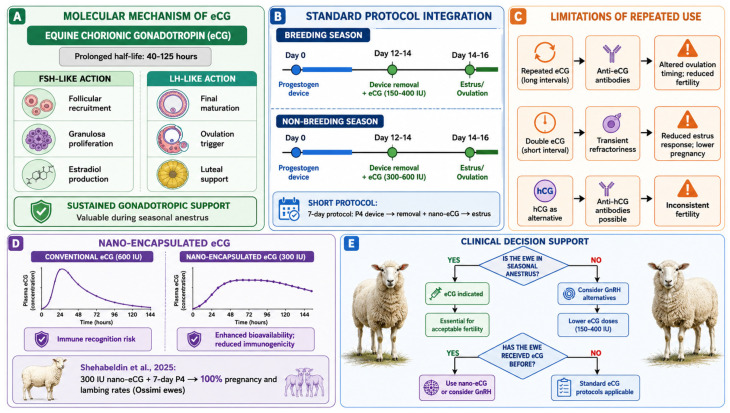
Schematic representation of eCG and hCG in ovine estrus synchronization. (**A**) illustrates the dual FSH/LH activity of eCG and its prolonged half-life (40–125 h), provides sustained gonadotropic support essential during seasonal anestrus. (**B**) depicts standard protocol integration: progestogen priming (12–14 days) followed by eCG at device removal, with breeding season requiring lower doses (150–600 IU) and non-breeding season requiring higher doses (300–600 IU). Short-term (7-day) progesterone protocol with eCG is also illustrated. (**C**) outlines limitations: anti-eCG antibody formation with repeated long-interval administration, transient refractoriness following short-interval double exposure, and inconsistent fertility outcomes with hCG as an alternative. (**D**) illustrates emerging nano-encapsulated eCG technology: sustained release enhances bioavailability and reduced immunogenicity, achieving 100% pregnancy and lambing rates with 300 IU in Ossimi ewes [50]. (**E**) presents a clinical decision framework based on seasonal status and prior eCG exposure. eCG: equine Chorionic Gonadotropin, FSH: Follicle-Stimulating Hormone, FSHR: Follicle-Stimulating Hormone Receptor, GnRH: Gonadotropin-Releasing Hormone, GnRH: gonadotropin-releasing hormone, hCG: human Chorionic Gonadotropin, IU: International Units, LH: Luteinizing Hormone, P4: Progesterone.

Progesterone-eCG combinations remain the most reliable cross-season protocol [47]. Less commonly, human chorionic gonadotropin (hCG) has emerged as an alternative for eCG. However, repeated administration may induce anti-hCG antibodies, potentially altering ovulation timing and reducing fertility [48]. A short-interval double eCG exposure has been associated with reduced estrus response and pregnancy rates, suggesting transient refractoriness [49]. The molecular mechanisms, protocol integration, and clinical considerations associated with eCG and hCG administration in ovine synchronization programs are illustrated in Figure 2. Effective doses typically range from 150 to 600 IU, with lower doses often sufficient following adequate progestogen priming [33,50]. Beyond clinical pregnancy outcomes, gonadotropic stimulation also influences follicular development, luteal competence, embryo quality, and ovarian receptor expression. The principal biological and ovarian consequences associated with hormonal synchronization protocols are summarized in Table 1. Nanotechnology-based delivery systems represent an emerging area of investigation. In a preliminary study in Ossimi ewes, 300 IU of nano-encapsulated eCG administered within a 7-day progesterone protocol resulted in 100% pregnancy and lambing rates, compared with lower rates achieved using 600 IU conventional eCG [50]. These findings suggest that sustained release formulation may enhance bioavailability and reduce immune recognition. However, these results derive from a single study and require validation across different breeds, management systems, and seasonal conditions before broader application can be recommended.

### 3.3. GnRH and Analogs

**Figure 3:**
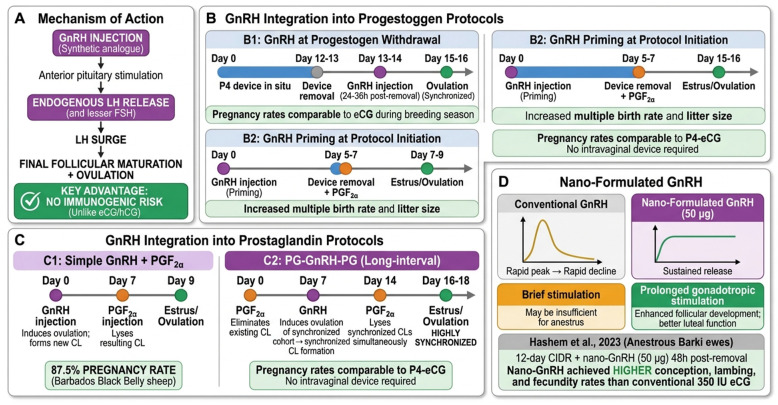
Schematic representation of GnRH and analogs in ovine estrus synchronization. (**A**) illustrates the physiological mechanism: GnRH stimulates endogenous LH release, inducing an LH surge that triggers final follicular maturation and ovulation. The key advantage over eCG/hCG is the absence of immunogenic complications. (**B**) depicts GnRH integration into progestogen protocols. (**B1**) standard administration at progestogen withdrawal (24–36 h post-removal) yielding pregnancy rates comparable to eCG during the breeding season. (**B2**) GnRH priming at protocol initiation increases multiple birth rates and litter size. (**C**) presents GnRH integration into prostaglandin protocols. (**C1**) simple GnRH + PGF_2_α achieves 87.5% pregnancy rates. (**C2**) PG-GnRH-PG protocol produces tighter estrus synchrony and pregnancy rates comparable to P4-eCG during breeding season without intravaginal devices. (**D**) highlights nano-formulated GnRH technology: sustained release provides prolonged gonadotropic stimulation, achieving superior conception rates to conventional eCG in anestrous ewes [57]. CL: Corpus luteum, eCG: equine Chorionic Gonadotropin, FSH: Follicle-Stimulating Hormone, GnRH: Gonadotropin-Releasing Hormone, hCG: human Chorionic Gonadotropin, LH: Luteinizing Hormone, µg: microgram, P4: Progesterone, PG: Prostaglandin, PGF_2_α: Prostaglandin F_2_ alpha.

GnRH induces an endogenous LH surge, promoting final follicular maturation and ovulation without the immunogenic risks associated with hCG/eCG [5]. It is particularly effective during the breeding season, yielding comparable pregnancy rates to eCG-based protocols when administered at device removal or 24–36 h later [55]. Incorporation of GnRH into prostaglandin-based systems has improved synchronization precision [5,56]. The long-interval PG-GnRH-PG protocol, involving PG on day 0, GnRH on day 7, and PG on day 14, induces formation of a synchronized corpus luteum that is subsequently lysed, resulting in tighter estrus synchrony and pregnancy rates comparable to P4-eCG protocols during the breeding season [5]. Simpler GnRH + PGF_2_α combinations have also achieved high pregnancy rates (87.5%) in Barbados Black Belly sheep [56]. The endocrine mechanism of GnRH action and its integration into both progestogen- and prostaglandin-based synchronization protocols are summarized in Figure 3.

Priming GnRH at the beginning of a short-term progestogen protocol has been shown to increase multiple birth rate and litter size compared to protocols without GnRH [33]. Nonetheless, GnRH-based approaches may induce shorter behavioral estrus and are less effective during deep seasonal anestrus due to limited follicular steroidogenic capacity [57,58]. In a preliminary study, nano-formulated GnRH demonstrated enhanced conception and fecundity rates compared to conventional eCG during anestrus in Barki ewes, likely due to sustained-release properties that mimic prolonged gonadotropic stimulation [57]. These promising results warrant further investigation across larger sample sizes and diverse breed types to confirm efficacy and safety.

### 3.4. Prostaglandin F_2_α-Based Systems

PGF_2_α and synthetic analogs such as cloprostenol induce luteolysis but are effective only in cycling ewes with a mature corpus luteum (≥day 5 post-ovulation), restricting their use to the breeding season [59]. The variability in estrus onset and ovulation following PGF_2_α administration primarily reflects the physiological heterogeneity in ovarian follicular status at the time of treatment. In cycling ewes, the corpus luteum is sensitive to the luteolytic action of PGF_2_α only after day 5 post-ovulation [59]. However, even among ewes with a functional corpus luteum, the stage of the follicular wave at the time of PGF_2_α administration varies considerably [26]. The time from luteolysis to ovulation, and consequently the interval to estrus, depends on the developmental status of the dominant follicle at the time of luteal regression [60]. Ewes in which luteolysis occurs in the presence of a mature dominant follicle will ovulate within a predictable window of 48–72 h [60,61]. In contrast, ewes with a small or absent dominant follicle require additional time for follicular recruitment and maturation, leading to ovulation intervals that may extend to 96 h or more [60,62]. This broad 48 to 120 h range in ovulation timing, often referred to as the estrus window, is a direct consequence of asynchronous follicular status across ewes. Consequently, the wide estrus window compromises the precision of FTAI, as the optimal insemination time relative to ovulation cannot be predetermined with sufficient accuracy [60].

**Figure 4:**
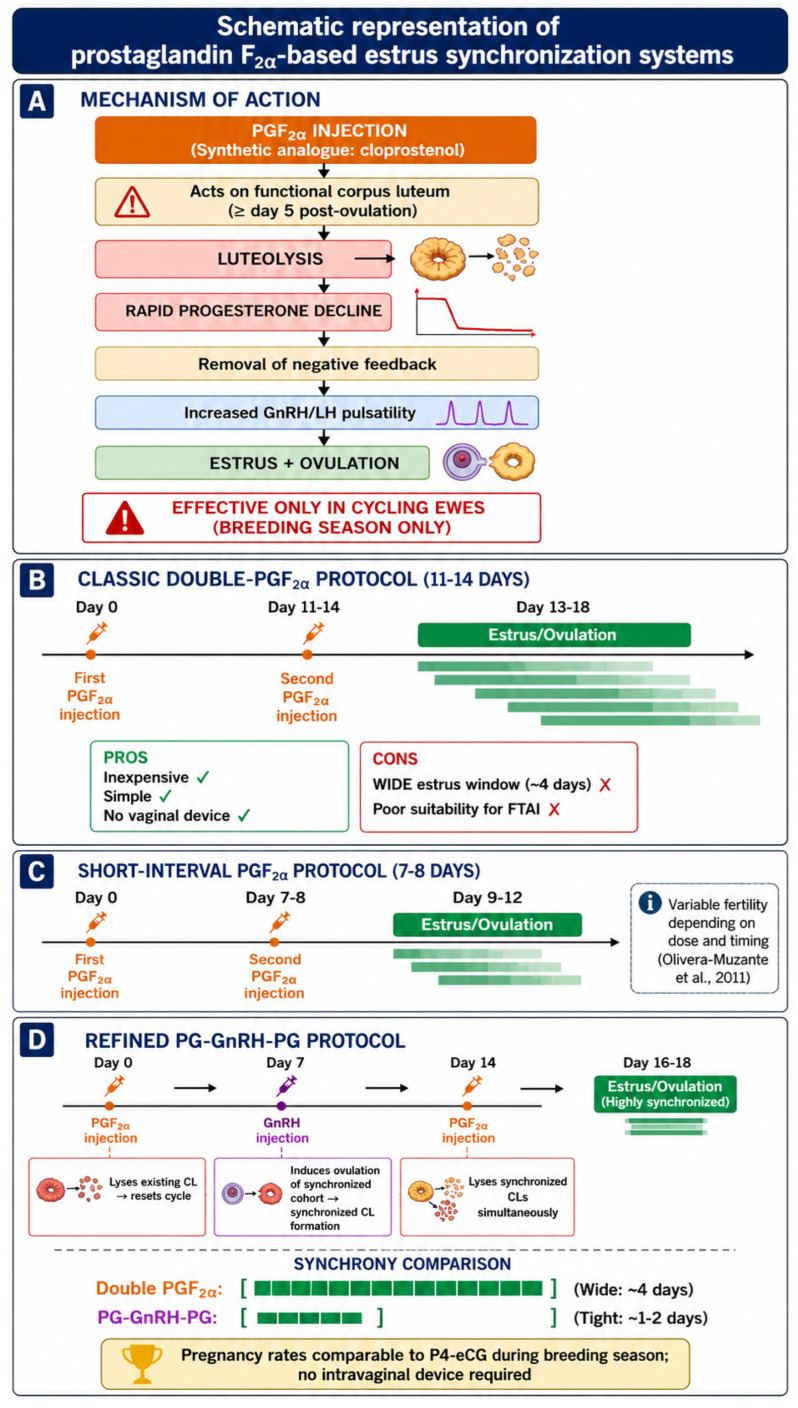
Schematic representation of prostaglandin f_2_α-based estrus synchronization systems. (**A**) illustrates the mechanism of action: PGF_2_α induces luteolysis by acting on a functional corpus luteum (≥ day 5 post-ovulation), causing rapid progesterone decline, removal of negative feedback, increased GnRH/LH pulsatility, and subsequent estrus and ovulation. The critical constraint is that PGF_2_α is effective only in cycling ewes during the breeding season. (**B**) Timeline for classic double-PGF_2_α protocol (11–14 days apart), which is inexpensive and simple but produces a wide estrus window (~4 days), limiting suitability for FTAI. (**C**) depicts the short-interval PGF_2_α protocol (7–8 days between injections), with variable fertility outcomes depending on dose and timing [63]. (**D**) presents the refined PG-GnRH-PG protocol [5]: PGF_2_α on day 0 eliminates existing corpora lutea; GnRH on day 7 induces ovulation of a synchronized follicular cohort, creating synchronized corpora lutea; PGF_2_α on day 14 lyses these synchronized corpora lutea simultaneously; all ewes then enter the follicular phase at the same time, resulting in highly synchronized estrus and ovulation. The visual representation compares synchrony width: double PGF_2_α produces a wide estrus window (approximately 4 days, unsuitable for fixed-time AI), whereas PG-GnRH-PG produces a tight window suitable for FTAI, with pregnancy rates comparable to progesterone-eCG protocols during breeding season. CL: Corpus luteum, FTAI: Fixed-Time Artificial Insemination, GnRH: Gonadotropin-Releasing Hormone, LH: Luteinizing Hormone, P4: progesterone, PG: Prostaglandin, PGF_2_α: Prostaglandin F_2_ alpha.

The classic double-PGF_2_α protocol (11–14 days apart) is inexpensive but produces a wide estrus window of approximately four days, limiting suitability for FTAI [5]. Refinements such as the PG-GnRH-PG protocol improve synchrony by coordinating follicular wave emergence and luteal regression [5]. Shorter intervals between PG injections (7–8 days) have been evaluated, with variable fertility outcomes depending on dose and timing [63]. The physiological mechanism of PGF_2_α-induced luteolysis and the architecture of the principal prostaglandin-based synchronization systems are illustrated in Figure 4.

Regulatory considerations also influence the practical applicability of prostaglandin-based protocols. In the European Union, while PGF_2_α analogs such as dinoprost are permitted for intramuscular administration in large animals, sheep is not mentioned as target species, and withdrawal periods is not officially indicated. Veterinarians and producers must therefore observe route-specific authorizations, as certain hormonal substances have been restricted to specific methods of administration based on residue considerations. These regulatory requirements, together with the limitation that PGF_2_α is effective only in cycling ewes, constrain the widespread use of prostaglandin-only protocols in commercial sheep operations.

A comparative overview of the principal synchronization protocols, including hormonal components, seasonal applicability, reproductive outcomes, and major advantages and limitations, is presented in Table 2.

## 4. Recombinant and Emerging Agents

**Figure 5:**
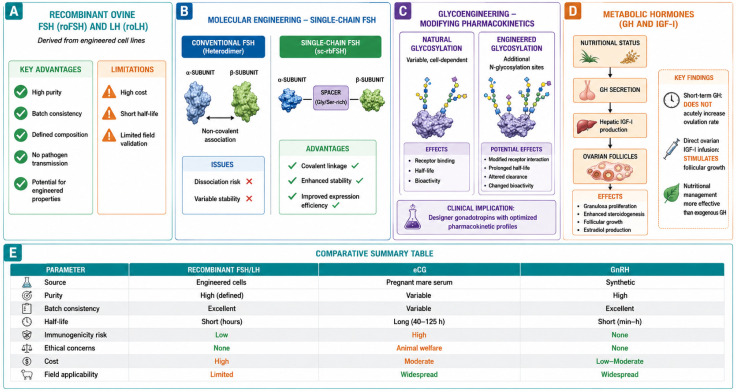
Schematic representation of recombinant and emerging agents in ovine reproductive management. (**A**) outlines recombinant ovine FSH (roFSH) and LH (roLH) derived from engineered cell lines, listing key advantages (high purity, batch-to-batch consistency, defined composition, no pathogen transmission risk, potential for engineered properties) and current limitations (high cost, short half-life, limited field validation). (**B**) illustrates molecular engineering of single-chain recombinant FSH: conventional FSH exists as a heterodimer with non-covalently associated α and β subunits, whereas single-chain recombinant bovine FSH (sc-rbFSH) fuses α and β subunits via a flexible glycine/serine-rich spacer peptide, preserving biological functionality while enhancing molecular stability and expression efficiency. (**C**) depicts glycoengineering strategies: natural glycosylation affects receptor binding, half-life, and bioactivity; engineered addition of N-glycosylation sites can modify receptor interaction, prolong circulatory half-life, alter clearance rate, and change bioactivity. (**D**) illustrates metabolic hormone pathways: nutritional status modulates GH secretion, which stimulates hepatic IGF-I production; IGF-I acts on ovarian follicles to promote granulosa cell proliferation, steroidogenesis, follicular growth, and estradiol production. Key findings indicate that short-term GH does not acutely increase ovulation rate, whereas direct ovarian IGF-I infusion stimulates follicular growth. (**E**) provides a comparative summary of recombinant gonadotropins, eCG, and GnRH across key parameters. eCG: equine Chorionic Gonadotropin, FSH: Follicle-Stimulating Hormone, GH: Growth Hormone, GnRH: gonadotropin-releasing hormone, h: hours; IGF-I: Insulin-like Growth Factor I, LH: Luteinizing Hormone, min: minutes, rbFSH: recombinant bovine FSH, roFSH: recombinant ovine FSH, roLH: recombinant ovine LH.

Emerging molecular and biotechnological approaches to ovine reproductive management, including recombinant gonadotropins and metabolic regulators of follicular function, are summarized in Figure 5. Recombinant ovine FSH and LH derived from engineered cell lines offer high purity and batch consistency [64,65]. Single-chain recombinant bovine FSH has been constructed by genetically fusing the α- and β-subunits via a flexible spacer peptide, preserving biological functionality while enhancing molecular stability and expression efficiency [65,66]. Incorporation of additional N-glycosylation sites may modify receptor interaction and circulatory half-life [67]. Although promising for controlled ovarian stimulation, cost and short half-life currently limit widespread field use. Metabolic hormones such as GH and IGF-I influence follicular dynamics primarily through nutritional modulation rather than direct ovarian stimulation. While short-term GH administration does not acutely increase ovulation rate, direct ovarian IGF-I infusion stimulates follicular growth and estradiol secretion [68,69,70]. eCG has been used as an LH surrogate, but inconsistent fertility outcomes and altered follicular dynamics make it less reliable than GnRH [48]. The efficacy of synchronization protocols is strongly influenced by seasonality, breed-specific ovarian responsiveness, and physiological status. These contextual modulators of treatment success are summarized in Table 3.

## 5. Discussion

The effectiveness of estrus synchronization in sheep cannot be adequately evaluated using a single parameter, as each measure of reproductive success offers distinct information with inherent limitations [47]. Estrus expression rate is the most commonly reported parameter, but it only indicates behavioral response to hormonal treatment and does not guarantee ovulation or subsequent fertility [36]. Pregnancy rate, typically diagnosed by ultrasonography or progesterone measurement, reflects early embryonic survival but does not account for pregnancy losses occurring after diagnosis, nor does it capture litter size [71]. In sheep, embryonic and fetal losses between early diagnosis and parturition can be substantial; one study reported that approximately 19.9% of ewes experienced late embryonic loss, fetal loss, or both, and 21.2% of embryos or fetuses were lost from day 25 to term [71]. Potential offspring were lost throughout gestation, with losses ranging from 3.3% to 11.5% across different gestational periods [71]. Furthermore, early embryonic loss can be surprisingly high without an apparent problem at lambing, reaching up to 30% of conceptions [71]. Lambing rate (number of ewes lambing per treated ewe) and fecundity (number of lambs born per treated ewe) encompass the entire reproductive process from ovulation through embryonic and fetal development to parturition, and are thus more direct indicators of productive and economic efficiency [72]. Discrepancies between pregnancy rate and lambing rate can arise from early embryonic mortality, fetal loss due to nutritional deficiencies or infectious diseases, neonatal mortality, and management factors such as dystocia or inadequate colostrum supply. Therefore, while estrus expression and pregnancy rates are useful for assessing specific stages of the reproductive process, fecundity provides the most integrative measure of synchronization success, reflecting both ovarian response and the ability of the ewe to sustain pregnancy through to term. This is particularly relevant for producers whose primary economic interest is the number of lambs born and weaned per ewe. Consequently, protocols that maximize estrus behavior or ovulation do not necessarily produce the highest fertility, whereas simpler protocols aligned with physiological timing can yield equal or superior pregnancy and lambing rates [36]. Fecundity, defined as the number of lambs born per treated ewe, represents a more integrative indicator of reproductive and economic efficiency [73].

Progesterone-based protocols, particularly when combined with eCG, consistently produce high estrus response (exceeding 85% in most studies) and conception rates, especially during seasonal anestrus. Their efficacy stems from two mechanisms: (i) luteal-phase simulation through progesterone priming, which establishes a uniform endocrine baseline and prevents premature LH surges, and (ii) gonadotropic support via eCG, providing potent FSH- and LH-like activity to recruit follicles and enhance ovulatory competence [36]. For instance, 12-day intravaginal progesterone sponges supplemented with 200 IU eCG achieve estrus response and conception rates of 92.86% and 84.61%, respectively, outperforming protocols using GnRH or insulin as adjuncts [74]. However, prolonged progestogen exposure can lead to persistent dominant follicles with altered steroidogenic profiles, potentially compromising oocyte quality and embryonic development [75]. The comparable or superior fertility achieved with short-term progestogen protocols reflects the physiological principle that ovulation of newly recruited follicle cohorts, rather than aged persistent follicles, optimizes oocyte quality and embryonic survival [75,76,77]. This observation supports the trend toward shorter treatment durations in modern synchronization programs, provided that luteolysis is adequately ensured through concomitant PGF_2_α administration in cyclic ewes. Repeated hormonal stimulation in pre-synchronization protocols may diminish ovarian responsiveness, thereby reducing estrus expression and FTAI success [49].

GnRH-based protocols highlight the physiological distinction between behavioral estrus and ovulatory competence. Ovulation can be tightly induced without overt estrus behavior, which is particularly relevant for fixed-time AI (FTAI) systems [78,79]. GnRH administration at 36 h post-progestogen withdrawal significantly reduces the CIDR-removal-to-ovulation interval (63.8 ± 1.38 h to 55.5 ± 0.48 h) and decreases variation in ovulation timing by approximately 62%, thereby improving synchrony [55]. This precision explains why progesterone + GnRH protocols can achieve acceptable pregnancy rates in FTAI despite reduced estrus expression. The success of prostaglandin-GnRH-prostaglandin protocols in achieving pregnancy rates comparable to progesterone-eCG regimens [5] highlights the potential for device-free synchronization systems during the breeding season. This approach is particularly attractive where intravaginal devices are impractical or where animal welfare concerns favor injectable-only protocol. Nevertheless, the effectiveness of GnRH is reduced during deep seasonal anestrus, when follicular steroidogenesis and LH pulsatility are suppressed. Under these conditions, eCG supplementation may be more effective in promoting follicular maturation and estrus expression [55,78,79].

Maximizing ovulation rate does not necessarily optimize reproductive efficiency. eCG-based protocols frequently increase ovulation rate and litter size, but excessive follicular stimulation can disrupt the synchrony between oocyte maturation and ovulation, reducing embryo survival [51]. Premature luteal regression has been documented in eCG-treated ewes, compromising progesterone support and embryo recovery [52]. CIDR-based protocols in tropical hair sheep have demonstrated earlier luteolysis and lower progesterone concentrations during the peri-implantation period compared with PG-only regimens, emphasizing the importance of luteal competence for successful pregnancy establishment [1]. Breed-specific variation is also critical. Breed-specific differences in gonadotropin responsiveness underscore the genetic modulation of ovarian receptor expression and follicular sensitivity to exogenous hormones [18]. These findings have practical implications for protocol optimization, suggesting that breeds with inherently higher responsiveness may benefit from reduced hormone doses to avoid excessive stimulation and its associated negative effects on embryo quality. Exogenous gonadotropins upregulate ovarian FSHR, LHR, and GnRHR expression in a dose-dependent manner, further influencing follicular responsiveness [54]. Moderate stimulation combined with adequate luteal support appears optimal, as demonstrated by protocols combining eCG with bovine somatotropin (bST). In Pelibuey ewes synchronized with FGA sponges, treatment with bST (125 mg, 5 days before sponge removal) combined with eCG (300 IU at sponge removal) resulted in a 100% conception rate, compared with 66% for eCG alone, 83% for bST alone, and 65% in the control group [53]. The proportion of ewes with multiple births was 80% in the bST + eCG group, compared with 69% for eCG alone, 35% for bST alone, and 20% in the control group [53]. Importantly, the embryonic survival rate was not compromised by the combined treatment; rather, bST enhanced serum IGF-I concentrations, which promote follicular development, fertilization, and embryonic survival [53]. These findings demonstrate that bST supplementation augments the prolificacy-enhancing effects of eCG without increasing embryonic loss, likely through IGF-I-mediated improvement of oocyte quality and embryo development [80].

Breed characteristics and seasonal reproductive activity strongly influence synchronization outcomes, often more than protocol design itself. Highly seasonal breeds, such as Mouton Vendéen and Ile-de-France, require stronger endocrine stimulation due to deeper seasonal anestrus and reduced LH pulse frequency [47,79,81]. In contrast, less seasonal or highly prolific breeds may achieve satisfactory fertility with lower hormone doses or GnRH-based protocols during the breeding season [82]. Even within the same breed, fecundity may vary under identical eCG protocols, indicating that genetic and physiological variability must be considered when designing synchronization programs [5,83]. These findings highlight the importance of adapting protocols to breed physiology, reproductive season, and ovarian sensitivity rather than relying on universal approaches [81,82]. The breed-specific differences in seasonality have direct implications for protocol design. Breeds with deep seasonal anestrus, such as the Suffolk, may require higher eCG doses or GnRH-based protocols to achieve acceptable fertility outside the breeding season, whereas less seasonal breeds such as the Chios or OOS line may respond adequately to simpler or lower-dose regimens. This variability underscores the importance of tailoring synchronization protocols to breed characteristics rather than applying a universal approach.

Beyond breed-specific genetic variation, a distinction between production types is clinically relevant. Intensively managed dairy breeds often experience a significant metabolic load during early lactation, which can compromise ovarian function and luteal competence, thereby modulating the endocrine environment established by exogenous progestogens and gonadotropins [70,82]. In such systems, synchronization success is closely linked to body condition score and nutritional management. In contrast, extensively managed meat breeds typically face the primary challenge of overcoming deep seasonal anestrus, with cost-effectiveness and field practicality being paramount considerations. While the present review focuses on sheep, it is noteworthy that analogous distinctions have been extensively documented in cattle, where intensive dairy and extensive beef breeds exhibit divergent responses to synchronization protocols; such findings underscore the broader principle that breed-type and production objectives must inform protocol selection. However, detailed comparative analysis across cattle production systems lies beyond the scope of this review.

Intravaginal synchronization devices may cause vaginal inflammation and alterations in the reproductive microbiota, particularly when long-term fluorogestone acetate (FGA) sponges are used, which can reduce fertility compared with CIDRs or short-term treatments [36,84]. Recent mitigation strategies include the use of Lactobacillus probiotics, which reduce neutrophil infiltration and may improve reproductive outcomes [85]. Ethical concerns regarding the production of eCG have also stimulated research into alternative technologies. Human recombinant FSH has demonstrated lambing and prolificacy rates comparable to conventional eCG, offering a non-animal-derived and potentially less immunogenic option [86]. The promising results obtained with nano-encapsulated gonadotropin formulations [50,57] demonstrate that sustained-release delivery systems can enhance biological efficacy at reduced doses, potentially mitigating both immunogenicity and cost barriers. However, these findings remain preliminary and require validation across diverse breeds and management systems. However, comparisons among studies remain difficult due to methodological heterogeneity, including differences in estrus detection methods, AI timing, semen type, and outcome definitions. Greater standardization of experimental design and reporting is therefore needed to enable robust comparisons and evidence-based protocol optimization [4,87].

## 6. Conclusions

Estrus synchronization in sheep has evolved from a purely hormonal intervention into a physiologically informed reproductive management strategy. Progesterone-based protocols, particularly when combined with eCG, remain the most consistent across breeds and seasons because they provide luteal priming and controlled follicular recruitment. However, accumulating evidence indicates that fertility depends more on follicular competence, ovulatory synchrony, and adequate luteal function than on maximizing estrus expression or ovulation rate alone. This explains the comparable reproductive outcomes achieved with short-term progesterone protocols and the strategic use of GnRH-based systems in fixed-time artificial insemination (FTAI) programs. Future progress should prioritize reducing dependence on eCG through the development of recombinant gonadotropins and precision endocrine modulation while tailoring synchronization protocols to breed characteristics and seasonal physiology. Emerging technologies, including nano-formulated hormone delivery systems, show preliminary promise but require rigorous validation across diverse production systems before clinical adoption. Practically, this means that protocol selection must account not only for physiological principles but also for the specific production context, intensively managed dairy flocks versus extensive meat flocks, as these systems differ markedly in metabolic status, seasonality, and management constraints. In parallel, greater attention to animal welfare, regulatory constraints, and standardized reporting of reproductive outcomes will be essential to support evidence-based protocol optimization. Such integrative approaches will facilitate the development of synchronization strategies that improve reproductive efficiency while meeting emerging ethical and sustainability expectations in sheep production systems.

## Figures and Tables

**Table 1:** Biological and ovarian consequences of hormonal synchronization protocols.

Biological Parameter	Observed Effect	Hormonal Context	Reproductive Implication	Reference
Non-fertilized oocytes	Higher with eCG (6.3 ± 2.4) vs. FSH only (0.8 ± 0.4)	pFSH ± eCG	Excess stimulation may reduce fertilization efficiency	[51]
Transferable embryos	Reduced with eCG (2.6 ± 1.1) vs. FSH only (5.8 ± 1.1)	pFSH ± eCG	Possible negative impact on embryo quality	[51]
Premature luteal regression	40% incidence in eCG-treated ewes	PGF_2_α + gonadotropin	Compromised embryo recovery	[52]
Luteal progesterone concentrations	Lower P4 (d7-13) in CIDR+PG vs. double PG	Progesterone vs. PG systems	Earlier luteolysis may affect luteal competence	[1]
Prolificacy	69% multiple births with eCG vs. 20% control; 80% with eCG+bST	FGA + eCG ± bST	eCG enhances ovulation rate; metabolic modulation improves fecundity	[53]
Conception rate	66% eCG vs. 100% eCG+bST	FGA + eCG ± bST	Synergistic metabolic-gonadotropic effect	[53]
Receptor expression	Upregulation of FSHR, LHR, GnRHR (dose-dependent)	eCG in vitro maturation	Enhanced follicular responsiveness	[54]

**Table 2:** Estrus synchronization protocol architectures in sheep: hormonal components and clinical performance.

Protocol	Hormonal Components (Typical Dose)	Season Applicability	Estrus and Fertility Outcomes	Major Advantages	Major Limitations
Long-term P4 + eCG	CIDR/FGA/MAP (12–14 d) + eCG (300–600 IU)	Breeding and Anestrus	Pregnancy 59–90%; high prolificacy	Reliable; effective during anestrus; strong ovulatory response	Persistent follicles; vaginal irritation; eCG immunogenicity
Short-term P4 + eCG	CIDR/FGA (5–7 d) + eCG (300–400 IU) + PGF_2_α	Breeding and Anestrus	Estrus > 88%; conception ≥ 80%; often higher fertility than long-term	Reduced P4 exposure; improved follicular quality; lower infection risk	Requires precise luteolysis timing
P4 + GnRH	P4 (7–14 d) + GnRH (50–100 µg) at removal	Primarily Breeding	Comparable to P4+eCG in season; increased litter size with priming GnRH	Avoids eCG immunogenicity; suitable for timed AI	Reduced efficacy in deep anestrus
Double PGF_2_α	2 × PGF_2_α (e.g., 0.1 mg cloprostenol), 11–14 d apart	Breeding only (cycling ewes)	Wide estrus window (~4 d); lower TAI precision	Low cost; no vaginal device	Requires functional CL; poorer synchrony
PG-GnRH-PG	PG (d0) + GnRH (d7) + PG (d14)	Breeding only	Pregnancy rates comparable to P4 + eCG; tighter synchrony; improved luteal P4	Injectable-only; strong synchrony; good luteal function	Three injections; cycling animals required
Long-interval PG + eCG	PG + eCG support	Breeding	Higher fecundity than PG alone; lower pregnancy vs. P4 + eCG (62.5% vs. 76.4%)	Device-free; moderate cost	Less effective than P4-based systems
Nanoparticle eCG (experimental)	P4 (7 d) + nano-eCG (300 IU)	Tested in breeding	100% pregnancy/lambing in a single preliminary study	Reduced dose; sustained release; possible lower immunogenicity	Experimental; cost and scalability unknown

**Table 3:** Seasonal and breed-dependent modulation of estrus synchronization outcomes.

Modulating Factor	Protocol Context	Observed Outcome	Interpretation	Reference
Seasonal anestrus	P4 + eCG	Pregnancy ~59% vs. 90% in breeding season	Reduced endogenous GnRH limits ovulatory competence	[4]
Deep anestrus	P4 + GnRH	Lower estrus expression and fertility vs. eCG	GnRH less effective without follicular steroidogenic readiness	[58]
Breed differences (chios, florina, karagouniko)	FGA + eCG	Fecundity: 99%, 85%, 95% respectively	Genetic modulation of gonadotropin sensitivity	[18]
Cycling status (cl ≥ day 5)	PGF_2_α-only protocols	Effective only in cyclic ewes	Physiological requirement for functional CL	[59]
Nutritional/metabolic status	eCG ± bST	Improved prolificacy and conception with metabolic support	IGF-I-mediated enhancement of follicular development	[53]

## Data Availability

No new data were created or analyzed in this study. Data sharing is not applicable to this article.

## Abbreviations

The following abbreviations are used in this manuscript:

CIDR	Controlled Internal Drug Release (intravaginal progesterone device)
CL	Corpus luteum
DNA	Deoxyribonucleic Acid
eCG	equine Chorionic Gonadotropin (formerly PMSG)
FSH	Follicle-Stimulating Hormone
FSHR	Follicle-Stimulating Hormone Receptor
FTAI	Fixed-Time Artificial Insemination
GH	Growth Hormone
GnRH	Gonadotropin-Releasing Hormone
hCG	human Chorionic Gonadotropin
IGF-I	Insulin-like Growth Factor I
LH	Luteinizing Hormone
P4	Progesterone
PG	Prostaglandin
PGF_2_α	Prostaglandin F_2_ alpha
PMSG	Pregnant Mare Serum Gonadotropin (former term for eCG)
